# Exploring the Potential Stimuli and Deterrents of Varicella-Zoster Viral Reactivation: A Scoping Review

**DOI:** 10.7759/cureus.81491

**Published:** 2025-03-31

**Authors:** Alexandra Lens, Blake Smith, Jessica Landi, Kristel Sibaja, Kaitlyn Pearl, Carly Snytte, Sukriti Prashar, Alexandria Sobczak, Brandon McConnell, Rohit Muralidhar, Michelle Demory, Marc Kesselman

**Affiliations:** 1 Medical School, Dr. Kiran C. Patel College of Osteopathic Medicine, Nova Southeastern University, Davie, USA; 2 Immunology, Dr. Kiran C. Patel College of Allopathic Medicine, Nova Southeastern University, Davie, USA; 3 Rheumatology, Dr. Kiran C. Patel College of Osteopathic Medicine, Nova Southeastern University, Davie, USA

**Keywords:** chickenpox, herpes zoster, postherpetic neuralgia, shingles, shingrix, vaccine, varicella, viral reactivation, zostavax

## Abstract

The varicella-zoster virus (VZV) causes a pruritic rash known as chickenpox during primary infection, which can become latent in neural tissue and reactivate as a painful rash called herpes zoster (HZ, also called shingles), often resulting from an age-related decline in cell-mediated immunity. A common complication following HZ is postherpetic neuralgia (PHN), a severe and often chronic neuropathic pain within the site of the initial HZ outbreak. The recombinant Shingrix and live attenuated Zostavax vaccines were created to provide immunity against viral reactivation. The objective of this scoping review is to evaluate the literature to understand the additional factors that contribute to VZV reactivation and can result in HZ and PHN. This review also aims to understand the vaccine efficacy (VE) of Shingrix and Zostavax in reducing VZV reactivation and PHN compared to non-vaccinated individuals. This study used the PubMed database to identify studies. Search terms included chickenpox, postherpetic neuralgia, reactivation stimuli, Shingrix, shingles, varicella zoster, "varicella zoster viral reactivation", and Zostavax. The exclusion criteria were literature reviews, meta-analyses, case reports, and gray literature. Only studies published in English between January 1, 2018, and April 1, 2023, were included. There were 17 studies extracted that focused on VZV reactivation stimuli, which indicated that comorbidities/disease stress, unrelated medical interventions, lifestyle/environmental assault, and depression were potential inciting factors of viral reactivation. There were two studies regarding the effect of body mass index (BMI) on HZ risk with conflicting results; one found a higher correlation of HZ with an overweight BMI, while the other found no correlation with a higher BMI and instead reported a higher HZ incidence in those with a normal BMI. There were 15 studies covering VE for HZ prevention and seven studies for VE against PHN, with some overlapping studies measuring both data values. Overall, the findings revealed that vaccinated individuals had consistently lower incidence rates of HZ and PHN compared to their non-vaccinated counterparts, as well as a consistently superior VE in Shingrix and a notable decline in VE with age. Three articles with data regarding PHN pathophysiology suggested that it is more likely caused by neurological damage with some genetic influence rather than further viral reactivation. While further investigation into the relationship between viral reactivation and risk factors is warranted based on this analysis, the results suggest that immunosuppression that has been previously linked to or correlated with these variables likely also contributes to VZV reactivation and PHN.

## Introduction and background

The varicella-zoster virus (VZV) is a contagious human herpesvirus characterized by a maculopapular, vesicular rash (known as chickenpox) that is extremely pruritic and lasts approximately 5-10 days [1]. Primary infection typically occurs in childhood, after which VZV establishes latency in the sensory and autonomic neurons in the dorsal or cranial root ganglia [1-3]. Although the host develops immunity against VZV, the virus can reactivate if the immune system becomes impaired. Reactivation of the latent virus can cause a painful, unilateral dermatomal rash termed herpes zoster (HZ), also known as shingles [4]. Most HZ cases resolve completely within one month of initial presentation, but between 10% and 20% of these cases can progress to a more chronic condition called postherpetic neuralgia (PHN) [5]. PHN is defined as the presence of extreme, chronic neuropathic pain lasting longer than 90 days after HZ onset that is localized in the region of the initial HZ outbreak. Patients may also experience abnormal sensory symptoms as well as mood disorders such as anxiety or depression [6]. PHN can last months to years or be lifelong. The condition has an overwhelmingly negative impact on patients' quality of life, particularly in older populations [7,8].

HZ was a common condition that affected over a million Americans each year before the advent of vaccines [7]. As of March 2025, two vaccines have been licensed to protect against HZ.

The live attenuated zoster vaccine (Zostavax, Merck & Co., Inc., Rahway, New Jersey) became available in the United States (US) in 2006, being the only approved HZ vaccine at the time. The vaccine contained the weakened form of the nonvirulent Oka strain of VZV that can induce an immune response without causing disease. According to the Shingles Prevention Study conducted by the Department of Veterans Affairs (VA), Zostavax reduced the incidence of HZ and PHN by 51.3% and 66.5%, respectively [9]. Apart from its limited efficacy, Zostavax was also contraindicated for immunocompromised patients and those taking biologic drugs; the vaccine was removed from the market in 2020.

The adjuvant recombinant zoster vaccine (Shingrix, GSK plc, London, UK) contains a recombinant glycoprotein E (gE) and AS01B Adjuvant System, consisting of a liposomal formulation of monophosphoryl lipid A (MPL), a toll-like receptor 4 (TLR4) agonist, and QS-21, a purified plant extract. The recombinant gE serves as the antigen portion of the vaccine, with the liposomes serving as carrier molecules to hold the antigen in place for immune response. The TLR4 agonist triggers the innate immune system and inflammatory cytokine response. The saponin adjuvant QS-21 stimulates a local transient innate immune response, which ultimately results in antigen-presenting cell recruitment and activation. QS-21 and MPL work synergistically to induce cytokine secretion such as interferon-gamma [2,8]. Overall, the vaccine components upregulate inflammation to elicit a broad immune response, including CD4 T-cells, and can enhance the production of neutralizing antibodies against VZV-infected cells.

The Advisory Committee on Immunization Practice (ACIP) from the Centers for Disease Control (CDC) recommends the two-dose Shingrix vaccine in immunocompetent adults aged 50 and older. The ACIP also recommends the vaccine in those 19 years of age and older who are immunocompromised. The two doses may be separated by two to six months in the immunocompetent and one to two months in the immunocompromised [10].

The superior efficacy of the Shingrix vaccine compared with the Zostavax vaccine has been shown to be attributed to its increased gE and VZV-specific Th1 memory responses. Although both vaccines elicit gE-specific responses, the gE-specific T-cell response was found to be at least 10-fold higher in the Shingrix vaccine compared to Zostavax at peak memory response (PMR), defined as 30 days following Zostavax or 30 days following the second dose of Shingrix analyzed via FluoroSpot and flow cytometry [11]. In addition, T-cell memory responses, represented as gE-IL-2+ and VZV-IL2+ spot-forming cells (SFCs), significantly improved in those who had previously received the Shingrix vaccine one year prior. Compared to Zostavax, Shingrix has been observed to have an overall higher efficacy, be minimally affected by age, and provide longer-lasting protection. The efficacy of Zostavax is ~51% in those older than 60, whereas the efficacy of Shingrix is ~97% in those aged 50 and older and 89% in those aged 80 and older [11]. In addition, vaccine-induced immune responses decreased significantly in the years following a single Zostavax vaccination compared to being preserved for six to nine years following the Shingrix vaccination series [11]. Despite the advent of these vaccines, several studies have reported an increase in the incidence of HZ over the past two decades, possibly due to an aging population with underlying comorbidities [12].

The severity of HZ outbreaks and PHN tends to increase with age, as VZV reactivation is generally attributed to an age-related decrease in immune function [4,6,8]. However, other circumstances such as comorbidities, immunosuppressant medications, and environmental factors may influence the onset of HZ in younger or previously immunocompetent individuals. These conditions have not been fully established and thus warrant further review and analysis.

## Review

Objective

The objective of this scoping review was to assess the extent of the literature on the potential stimuli and deterrents of VZV reactivation, the incidence of reactivation after vaccination, the incidence of VZV and PHN in unvaccinated individuals, and to investigate the pathophysiology of PHN. Zostavax and Shingrix vaccines were thoroughly assessed to conclude their overall efficacy in preventing HZ and PHN.

Methods

The scoping review was conducted in accordance with the JBI methodology for scoping reviews [13]. A preliminary search of the MEDLINE database, the Cochrane Database of Systematic Reviews, and the JBI Evidence Synthesis database was conducted. No current or ongoing systematic reviews or scoping reviews on the topic were found.

The search strategy involved locating only published studies. An initial limited search of Google Scholar and PubMed was undertaken to identify articles on the topic. The text words contained in the titles and abstracts of relevant articles and the index terms used to describe the articles were used to develop a full search strategy for PubMed, an electronic database; this was the sole database used for this review (see Appendix I). No unpublished studies or gray literature were searched.

Following the search, all identified citations were collated and uploaded into Rayyan 2022 (Rayyan Systems Inc., Cambridge, MA), and duplicates were removed. Following a pilot test, titles and abstracts were then screened by nine independent reviewers, with a minimum of two reviewers per article for assessment against the inclusion criteria for the review. The full text of potentially relevant sources was assessed in detail against the inclusion criteria by at least two independent reviewers per article during the second screening. Any disagreements that arose between the reviewers at each stage of the selection process were resolved through discussion or with an additional reviewer(s). The citation details of the included articles were imported into EndNote 20.6 (Clarivate, London, UK) for full accessibility.

The study's search results, inclusion process, and rationale for exclusion are reported in full in a Preferred Reporting Items for Systematic Reviews and Meta-analyses extension for scoping review (PRISMA-ScR) flow diagram [14]. The inclusion criteria were studies published in English between January 1, 2018, and April 1, 2023. Exclusion criteria included case studies, studies with less than 10 participants, and studies with data from the chickenpox vaccine or any HZ vaccine besides the Zostavax or Shingrix vaccines. Participants aged 19 or older were considered in articles relating to theories of viral reactivation and PHN pathophysiology. Meanwhile, participants 50 years of age or older were considered for articles comparing vaccine efficacy (VE), HZ incidence, and PHN incidence in vaccinated versus non-vaccinated participants due to the vaccines' age restrictions. VE studies with epidemiological data, such as the HZ incidence rate that did not include stratifications regarding vaccination status (i.e., the incidence rate in vaccinated participants versus a placebo/control group), were excluded due to a lack of experimental comparison. Studies using any non-human participants or that did not provide sufficient information about the participants (i.e., no information on age) were also excluded. Studies that did not provide the participant age range but provided a mean age were included if the overall mean of all participants was greater than or equal to 50.

**Figure 1:**
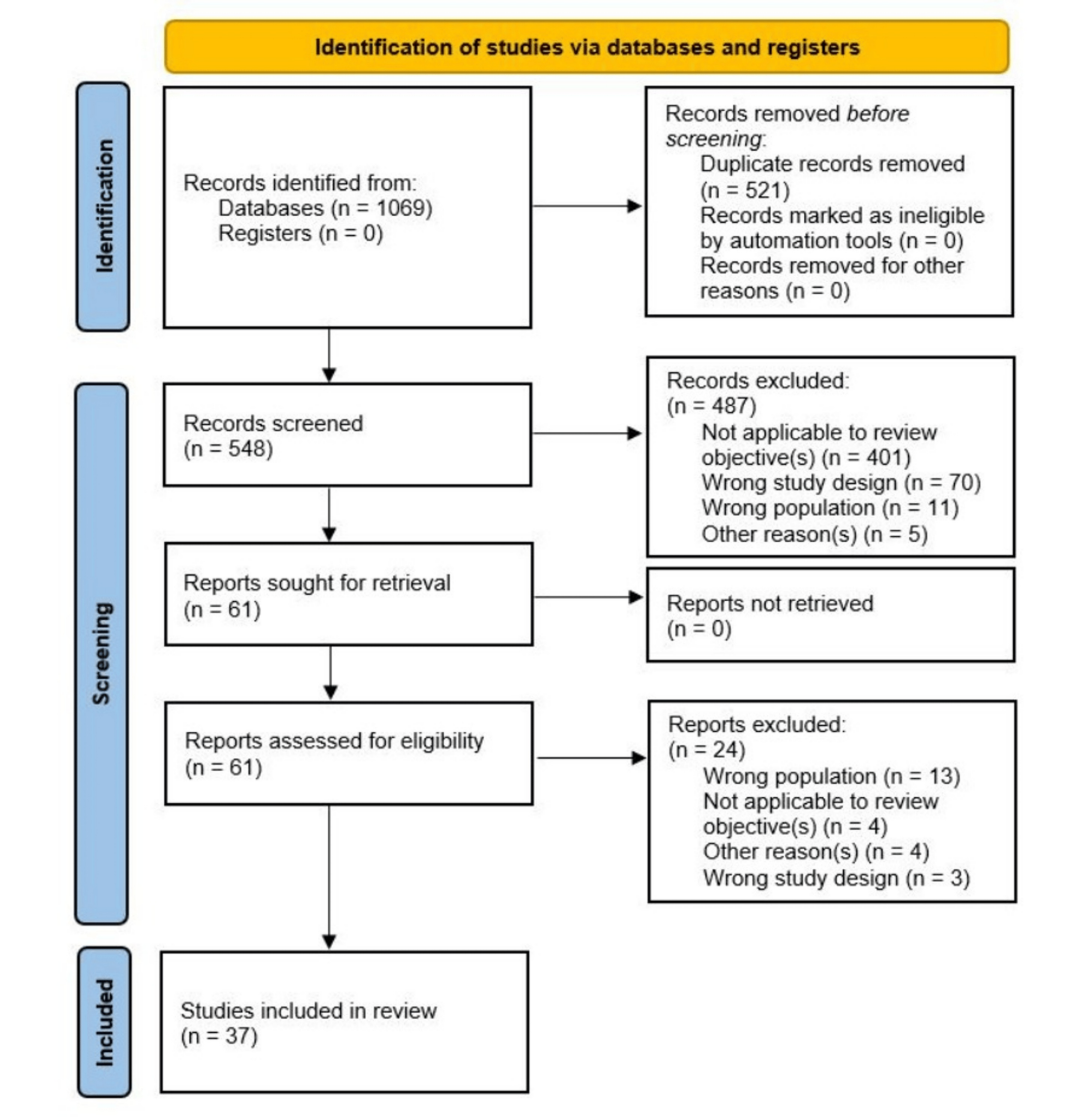
PRISMA flow diagram PRISMA: Preferred Reporting Items for Systematic Reviews and Meta-analyses

The unspecified "other reasons" for article exclusion in Figure 1 included non-scientific articles (e.g., a letter to an editor or opinion papers), insufficient information regarding the study participants (e.g., unknown mean age and age range), wrong outcome (e.g., no correlation found between two variables), or wrong sample size. Figure 1 delineates the review and elimination process of the articles found, explaining the number of articles excluded as well as the rationale behind these decisions for review Tiers I and II, respectively.

The data extraction as part of this review was modeled after the JBI SUMARI data extraction form for reviews [13]. Data were extracted manually from articles included in the scoping review by three independent reviewers. The data extracted includes specific details about the participants, concept, context, study design, stratifications, and key findings relevant to the review questions. Within these tables, the "Stratifications" column is defined as the further classification of data between two study cohorts using variables that may correlate with certain findings.

The draft data extraction tool was modified and revised as necessary during the process of extracting data from each included evidence source. The Participant/Concept/Context (PCC) framework was adapted into column categories such as "Source & Country," "Study Groups" (e.g., risk of HZ in patients with sciatica versus control group), "Study Design," "Study Size Number (n)," "Mean Age and/or Age Range of Participants," "Stratifications," "Type of Data Reported," and "Findings/Conclusions." The only notable distinction from the data extraction form was a separate column dedicated to the HZ or PHN incidence rate ratio of vaccinated to unvaccinated individuals rather than including the information in the "Findings/Conclusions" column. This was done to make these findings more accessible to view and interpret, as they directly address two objective questions of this scoping review.

Results

**Figure 2:**
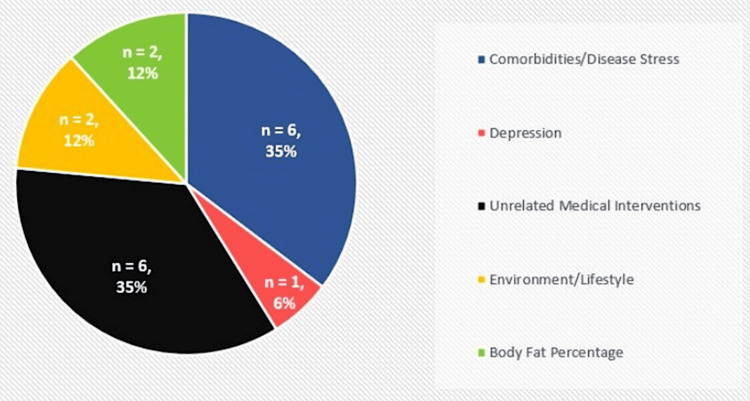
VZV reactivation stimuli summary of findings n: number of studies; VZV: varicella-zoster virus

The purpose of this scoping review was to analyze the existing literature on VZV reactivation stimuli, Zostavax/Shingrix VE against HZ and PHN, and the pathophysiology of PHN. This was achieved with data extracted from a total of 37 studies. Table 1 and Figure 2 below display data from the 17 articles focused on factors that contribute to VZV reactivation. These studies revealed a correlation between HZ outbreak and comorbidities/disease stress, unrelated medical interventions, body fat percentage, environment/lifestyle, and one study addressing depression as another proposed stimulus. While these variables may not be confirmed as direct causes of viral reactivation, they can at least be identified as potential contributing factors.

**Table 1:** Studies discussing/relating to theories and/or evidence of VZV reactivation stimuli ACoS: adhesive capsulitis of the shoulder; aHR: adjusted hazard ratio; anti-TNF: anti-tumor necrosis factor; BMI: body mass index; CCI: Charlson comorbidity index; CIC: chronic interstitial cystitis; CI: confidence interval; CMP: chondromalacia patella; DMARDs: disease-modifying antirheumatic drugs; DQS: de Quervain syndrome; HPFS: Health Professionals Follow-Up Study; HR: hazard ratio; HZ: herpes zoster; IR: incidence rate; IRR: incidence rate ratio; MVHR: multivariable-adjusted hazard ratio; NHS: Nurses' Health Study; PFF: plantar fascial fibromatosis; RA: rheumatoid arthritis; SpA: spondylarthritis; SEB: *Staphylococcus aureus* enterotoxin B; UVR: ultraviolet radiation; VZV: varicella-zoster virus

Source & Country	Study Group(s)	Study Design	Study Size Number (n)	Mean Age and/or Age Range of Participants	Stratification(s)	Type of Data Reported	Finding(s)/Conclusions
Kawahira et al., 2022 [15] Japan	Risk of HZ in people with normal BMI vs. underweight and overweight BMI (three independent variables)	Prospective cohort study	10,493	≥50 years	Gender, BMI classification (underweight, normal weight, overweight)	Prevalence, multivariate HRs, IR (per 1,000 person-years)	People of normal weight had the highest prevalence of HZ overall (15%) and in both genders, followed by overweight and underweight BMI in both genders. An overweight BMI was associated with a lower incidence of HZ than a normal weight BMI in older Japanese women.
Hsu et al., 2020 [16] Taiwan	Risk of HZ in patients with CIC vs. patients without CIC	Case cohort study	1,096 patients with CIC; 4,384 control group	≥20 years	Age, gender, comorbidities	IR (per 1,000 person-years)	The IR of HZ in patients with CIC was higher (10.8) than those who did not have the condition (7.25). HZ risk for the CIC cohort was 1.48 times that for the control cohort. In participants ≤49 years, patients with CIC had a 1.91-fold increased HZ risk compared to those without CIC.
Hsu et al., 2021 [17] Taiwan	Risk of HZ in patients with DQS vs. patients without DQS	Retrospective population-based cohort study	4,195 patients with DQS; 4,195 control group	≥20 years	Age, gender, comorbidities	IR (per 1,000 person-years)	The HZ IR in the DQS group was 8.39, and patients with DQS had a 1.30 times higher risk of HZ reactivation than the control group. DQS increases the HZ risk in individuals ≤64 years, women, and patients without comorbidities.
Schmidt et al., 2021 [18] Denmark	Risk of HZ in patients with several lifestyles/social habits vs. patients without these habits	Population-based cohort study	101,894	Age range ≥25 years; mean 58 years	Lifestyle factors	HR	There was no association of HZ with smoking, alcohol consumption, BMI, or level of physical activity, but there was a slight increase of hazard among former smokers (1.17, 95% CI: 1.06, 1.30) compared with individuals who had never smoked.
Chen et al., 2022 [19] Taiwan	Risk of HZ in patients with CMP vs. control group	Retrospective population-based cohort study	22,710 patients with CMP; 90,840 control group	≥20 years	Age, gender, comorbidities	IR (per 1,000 person-years) and HR	The HZ IR in the CMP group was higher (7.94) than in the control group (7.35). The CMP group also had a higher risk of HZ (aHR = 1.06, p < 0.05). Patients over age 65 years in the CMP group also had a higher risk of HZ than the control group (aHR=1.22, p<0.01). Women with CMP were at greater HZ risk than women in the control group (aHR=1.18, p <0.01).
Ke et al., 2022 [20] Taiwan	Risk of HZ in patients with sciatica vs. control group	Retrospective population-based cohort study	49,023 patients with sciatica; 49,023 control group	≥20 years	Age, gender, comorbidities	HR	Association found between sciatica and HZ risk, which was significantly higher in the sciatica cohort than in the control group (adjusted HR = 1.19; 95% CI = 1.12-1.25). Females were at a higher risk than males.
Wei et al., 2022 [21] Taiwan	Risk of HZ in patients before vs. after receiving Influenza vaccine	Population-based retrospective self-controlled case series	13,728	65.47 years	Age, gender, history of autoimmune disease, history of cancer, CCI score, number of days after vaccination	IRR for HZ (risk vs. control interval)	There was a slightly higher risk of HZ in patients 1-15 days after they received the influenza vaccine (IRR = 1.11; 95% CI, 1.02–1.20), but an insignificant risk for 1–30 days (IRR = 1.04; 95% CI, 0.98–1.10). The IRRs were much higher in participants aged 50–64 years old (1.16; 95% CI, 1.02–1.33), males (1.14; 95% CI, 1.01–1.28), and those with no history of cancer or autoimmune diseases.
Hsu et al., 2021 [22] Taiwan	Risk of HZ in patients with PFF vs. control group	Retrospective cohort analysis	4,729 PFF patients; 18,916 control group	≥21 years, mean 52.3 years in the PFF cohort, 51.9 years in the control cohort	Age, gender, comorbidities	HR and aHR	PFF patients were 1.23 times more likely to develop HZ than the control group. Male patients (aHR = 1.44) and patients aged ≥65 years (aHR = 1.48) in particular are at a higher risk of HZ.
Shimizuguchi et al., 2020 [23] Japan	Risk of HZ in cancer patients who received radiation therapy vs. patients who did not receive radiotherapy	Propensity score-matched, retrospective cohort study	4,350 cancer patients with radiotherapy matched with 4,350 non-radiotherapy patients	64.9 years in radiotherapy cohort, 65.4 years in non-radiotherapy cohort	Age, gender, primary malignant disease, initial metastatic status, comorbidity, treatment received, time since radiotherapy	HZ IR (per 1,000 person-years)	Cancer patients who received radiotherapy had an overall higher IR of HZ (11.7) than the non-radiotherapy group (4.3). Males, patients with positive metastatic status, and patients over age 70 had higher IRs in both cohorts. Among different types of malignancies/comorbidities, gastrointestinal malignancies and autoimmune diseases had the highest HZ incidence in the radiotherapy cohort, while malignant lymphomas and HIV had the highest HZ incidence in the non-radiotherapy cohort. Cancer patients who received surgery or systemic therapy both had a higher HZ incidence in the radiotherapy cohort.
Redeker et al., 2021 [24] Germany	HZ risk in patients receiving conventional synthetic, targeted synthetic, or biologic DMARDs	Prospective cohort study	13,991	57.7 years	Type of RA treatment	HZ event rate and IR (per 1,000 person-years), Exposure-adjusted event rates	The targeted synthetic DMARDs had the highest exposure-adjusted event rate (21.5, 95% CI 16.4 to 27.9) as well as a 3.6-fold increased risk of HZ associated with them, followed by B cell-targeted therapy (10.3, 95% CI 8.0 to 13.0), monoclonal anti-TNF antibodies (9.3, 95% CI 7.7 to 11.2), interleukin 6 inhibitors (8.8, 95% CI 6.9 to 11.0), soluble TNF receptor fusion protein (8.6, 95% CI 6.8 to 10.8), T-cell costimulation modulator (8.4, 95% CI 5.9 to 11.8) and conventional synthetic DMARDs. There was also an increased risk of HZ under biologic DMARDs compared to conventional synthetic DMARDs.
Choi et al., 2019 [25] Korea	HZ risk in individuals diagnosed with depression vs. matched control group	Longitudinal follow-up study	58,278 participants with depression; 233,112 control group	≥20 years	1476 levels (age (18 categories), gender (two categories), and income level (41 categories))	HR, aHR, and prevalence (%) for HZ in individuals with depression	HZ risk was higher overall in the depressed cohort (6.8% (3967/58,278)) than in the control cohort (6.3% (14,689/233,122), P < 0.001). The aHR was elevated only in women who were younger than 60 years of age (1.13 (95% CI: 1.02-1.25; P=0.16)) in women younger than 40 years of age and (1.11 (95% CI: 1.04-1.17; P<0.001)) in women aged 40-59 years.
Freuer et al., 2020 [26] Germany	Risk of HZ seropositivity in obese participants versus risk of seropositivity of other chronic infections in obese participants	Cross-sectional and longitudinal study (2006/08 and 2013/14 follow-up exams)	3,080 participants in first follow-up examination F4; 2,279 participants in the second follow-up examination FF4	56 years	Body mass index, body adiposity index, waist circumference, waist-to-hip ratio, and waist-to-height ratio	Seropositivity and the prospective occurrence of seropositivity for the VZV	Higher levels of body fat (regardless of fat distribution) were associated with a higher risk of VZV seropositivity in women, and higher body fat may be a risk factor for VZV reactivation.
Kawai et al., 2020 [27] United States of America	HZ risk in patients with varying levels of UVR exposure (based on updated geocoded address histories)	Three prospective cohort studies	205,756	Overall 50 years; 52 years in HPFS, 62 years in NHS, and 36 years in NHS II	Age and comorbidities	Incident HZ cases identified by self-reported clinician diagnosis	Ambient UVR exposure correlated with a higher risk of HZ in men (MVHR comparing highest vs. lowest quintiles: 1.14; 95% CI: 1.02, 1.29; P-trend=.03 in HPFS) but not in women (MVHR 0.99; 95% CI: 0.93, 1.05 in NHS and MVHR 0.96; 95% CI: 0.90, 1.03 in NHS II). A history of severe sunburn was associated with a moderately increased HZ risk in both men and women in all three cohort studies.
Schub et al., 2019 [28] International	Risk of HZ in RA or SpA patients receiving various antirheumatic drugs vs. healthy control group	Prospective cohort study	98 total participants; 78 RA patients; 20 SpA patients; 39 control group	60.6 years in patients with RA; 51.4 in patients with seronegative SpA; 58.6 years in healthy control group	Type of antirheumatic drug given (or combination therapy)	Expression of cytokines and cell surface markers of VZV-specific T-cells	RA patients had decreased CD4 cell expression (both VZV-specific and SEB-like) compared to the control group and thus showed signs of impaired cellular immunity. Patients treated with biological DMARDs had the most devastating impact on their cellular immunity.
Wollenhaupt et al., 2019 [29] International	HZ risk in RA patients receiving 5 mg tofacitinib twice daily vs. 10 mg tofacitinib twice daily	Global, open-label, long-term extension study	4481 total participants; 1,123 patients in the 5 mg cohort; and 3,358 patients in the 10 mg cohort	53.3 years overall; 54.0 years in the 5 mg group; 53.0 years in the 10 mg group	None	Number/percentage of adverse events among patient population, HZ IR (per 100 patient-years)	A higher dose of tofacitinib (10 mg) was associated with a higher percentage of HZ cases (11.5%) and an increased IR of HZ (3.36) compared to the 5 mg group (10.6% and 2.56, respectively). Overall IR was 3.13, and the percentage of HZ cases was 11.3%.
Hsu et al., 2020 [30] Taiwan	HZ risk in patients with ACoS vs. healthy control group	Retrospective cohort study	60,478 total patients; 30,239 ACoS patients; 30,239 control group	≥20 years	Age, gender, comorbidities	Number of HZ cases, HZ IR (per 1,000 person-years), crude HR, and aHR	Patients with ACoS had an overall 1.28 times higher risk of HZ than the healthy control group; each age group with ACoS had increased HZ risk, especially in patients younger than 50 years of age (aHR: 1.52, 95% CI: 1.31–1.75). Compared to the control groups, the HZ hazard ratio was higher for male patients (aHR: 1.40, 95% CI: 1.26–1.55) in the ACoS group than that for female patients (aHR: 1.22, 95% CI: 1.13–1.32). Participants with comorbidities, regardless of whether they had ACoS, had a lower risk of HZ (aHR: 1.16) compared to participants without comorbidities (aHR: 1.36).
Lee et al., 2021 [31] Taiwan	HZ risk in head and neck cancer patients who received radiotherapy vs. head and neck cancer patients who did not receive radiotherapy and healthy control group	Population-based cohort study	3,240 total participants; 1,080 radiotherapy patients; 1,080 non-radiotherapy cohort; 1,080 control group	≥30 years	Age, gender, comorbidities, use of other cancer treatments (oncological surgery and/or chemotherapy)	Number of HZ cases, HZ IR (per 1,000 person-years), crude HR, and aHR	HZ IR was higher in cancer patients than in non-cancer patients (13.67 vs. 8.06 per 1,000 person-years, p = 0.18) and in those who received radiotherapy than in those who did not (18.55 vs. 9.06 per 1,000 person-years, p = 0.03, respectively). RA was the only comorbidity that showed a significantly higher HZ risk (p = 0.02). Other cancer treatments, such as oncological surgery and chemotherapy, did not cause any significant impact on HZ onset.

Several comorbidities were associated with an increased risk and incidence of HZ, such as chronic interstitial cystitis, de Quervain syndrome, chondromalacia, sciatica, plantar fascial fibromatosis, and adhesive capsulitis of the shoulder [16,17,19,20,22,30]. A common trait of these conditions is chronic pain; six studies evaluated in this scoping review suggested that disease stress, particularly from chronic pain conditions, leads to higher HZ risk because it also increases the risk of depression, which is associated with reduced lymphocytic proliferative response and decreased T-cell proportion [19,22]. One study exploring the prevalence of HZ in depressed patients compared to a healthy control group found that patients with depression had a higher prevalence for HZ, with an increased HZ risk in female patients under 60 years of age [25]. Additionally, this study suggested that this phenomenon is a result of decreased cellular immunity observed in depressed patients [25]. Another study suggested that stress and pain cause changes within the human body's perceptual and stress systems, resulting in abnormal neuromatrix output patterns associated with decreased VZV-specific cellular immunity and subsequent viral reactivation [20].

Three studies investigated HZ risk in the presence of both an autoimmune disease and its respective immunosuppressive treatment. The medications that were studied include disease-modifying antirheumatic drugs (DMARDs), monoclonal anti-TNF antibodies, soluble TNF receptor fusion proteins, T-cell costimulation modulators, B cell-targeted therapies, IL-6 inhibitors, Janus kinase (JAK) inhibitors, corticosteroids, and glucocorticoids. One of these studies sought to compare HZ risk in patients with RA or SpA and how immunosuppressant medications impacted the risk level. It found that patients with RA, but not SpA, had an increased risk of HZ, which worsened if they were taking DMARDs to treat it, especially biologic DMARDs (bDMARDs) [28]. The second study compared the impact of various RA drugs on HZ risk in RA patients, which found that JAK inhibitors carried a significantly higher risk of HZ infection compared to other RA drugs; JAK inhibitors block intracellular cytokine signals, suppressing the body's immune response [24]. Biologic DMARDs also carried a much higher risk for HZ than conventional synthetic DMARDs [24]. Glucocorticoids had a dose-dependent increased HZ risk, with a 3.5-fold higher risk at doses exceeding 10 mg daily [24]. The third study compared the risk of HZ in RA patients taking a 5 mg twice daily dosage of tofacitinib, a JAK inhibitor, versus a 10 mg dose. The study revealed that patients who took the 10 mg dose had a higher incidence rate of HZ than the 5 mg cohort [29]. Vulnerabilities in the immune system caused by autoimmune conditions or immunosuppressant medications are thought to be responsible for premature or accelerated VZV reactivation. These patients may need prophylactic treatment for HZ to lessen the risk of this complication.

One study compared HZ risk in patients before and after receiving the influenza vaccine, further dividing the post-vaccination cohort into days 1-15 versus days 1-30 after receiving the vaccine [21]. The results found a higher incidence rate ratio (IRR) of HZ in the first 15 days post-vaccination compared to the other cohorts, with a particularly increased risk in males, patients 50 to 64 years of age, and patients with no significant medical history. The reason for this increased risk is unknown, although some hypothesize that it may be due to the mechanism of mRNA vaccines, such as the influenza vaccine, that induce immune reconstitution [21]. This could potentially serve as a baseline for future studies.

Two studies yielded conflicting results regarding the association between body fat percentage and VZV reactivation. A 2022 Japanese study compared HZ incidence in individuals categorized by BMI and found that individuals with a normal BMI (18.5-24.9 kg/m^2^) had a higher incidence rate of HZ compared to their underweight (<18.5) or overweight (≥25) counterparts in both genders, particularly older females [15]. However, this study used self-reported weight/height measurements at baseline rather than data values from the onset of HZ symptoms, which could have diminished existing associations [15]. There was also a significantly higher number of participants in the normal BMI category compared with the underweight and overweight classifications, which may have caused a higher recorded incidence rate of HZ outbreaks.

A German study published in 2020 focused on whether body fat distribution and obesity can influence the seropositivity of VZV by utilizing a multitude of anthropometric measures such as BMI, body adiposity index (BAI), waist-to-hip ratio, waist-to-height ratio, and waist circumference. The participants in this study were measured digitally by certified examiners, and considerations such as light clothing were noted. These measures allowed researchers to focus on both general and abdominal obesity. All of the indices, with the exception of the waist-to-hip ratio, showed a significant positive linear correlation between obesity in women and both the prevalence of seropositivity and the potential development of VZV seropositivity. It should be noted that this study included a broad definition of seropositivity beyond reactivated latent infection (i.e., primary infection, presence of antibodies from previous infection), which may have impacted the specificity of the results. Although further investigation found that associations are independent of adipose tissue distribution within individuals, the results supported the notion that total body fat positively correlates with primary infection or reactivation of latent VZV infection in women [26]. While the results from this study suggest a correlation between body fat and reactivation, the limitation of a few published studies necessitates further investigation.

Three studies found evidence of a correlation between radiation exposure and an increased risk of developing HZ in differing contexts. The first was a retrospective cohort study comparing HZ incidence in cancer patients who had received radiotherapy with cancer patients who did not (categorized under "Unrelated Medical Interventions" in Figure 2). The results revealed that cancer patients treated with radiotherapy had a significantly higher risk of developing HZ compared to non-radiotherapy patients [23]. The second study was similar, focusing on HZ risk in head and neck cancer patients who received radiotherapy versus those who did not; the study also sought to find any potential correlations between HZ risk and other treatment modalities such as oncological surgery and chemotherapy. The results again revealed that cancer patients who received radiotherapy had a higher risk of HZ than patients who did not receive radiotherapy (with no significant impact from other treatment modalities) [31]. The third study aimed to find any correlation between the level of lifetime ambient ultraviolet radiation (UVR) exposure with documented cases of HZ among three cohorts, which is categorized under "Environment/Lifestyle" in Figure 2. A higher lifetime exposure to UVR was associated with a higher risk of HZ in men but not in women. The reason for this was unclear, but the authors cited a prior study that revealed a higher susceptibility of UVR-induced immunosuppression in men than in women; they also speculated a gender difference in sun protection habits as another potential explanation. A higher frequency of sunburn was associated with higher HZ risk in all three cohorts [27]. This outcome was not surprising given that ionizing radiation, often coupled with its dermatologic side effects, is known to impair regional cellular immune response and immune monitoring, creating a higher likelihood of HZ outbreaks [23].

A 2021 Danish study investigated any possible link between social habits/lifestyle (smoking, alcohol consumption, etc.) and future HZ outbreaks. The only variable linked with a higher risk of HZ was a former history of smoking; however, current smokers did not have an observable correlation with HZ [18].

Table 2 displays data from 15 articles comparing the incidence of VZV reactivation in vaccinated individuals compared with their non-vaccinated counterparts. Although there was some variation in the extent of VE among these studies, the consensus was that vaccinated individuals had lower incidence rates of HZ than non-vaccinated individuals. Still, there was one study from Sweden that can be considered an outlier. The study estimated a relatively low overall VE (34%) from Zostavax, even reporting an overall higher HZ incidence rate in vaccinated participants than their unvaccinated counterparts. Only the cohort that included individuals aged 61-75 years showed a decreased HZ risk in vaccinated individuals [32].

**Table 2:** Studies analyzing VE and/or comparing the incidence of VZV reactivation in vaccinated versus non-vaccinated individuals aged 50+ CI: confidence interval; HZ: herpes zoster; LTFU: long-term follow-up; NA: not applicable; IR: incidence rate; IRR: incidence rate ratio; VE: vaccine efficacy; VZV: varicella-zoster virus

Source & Country	Study Group(s)	Study Design	Study Size Number (n)	Mean Age and/or Age Range of Participants	Stratification(s)	Type of Data Reported	Overall IRR (Vaccinated to Non-vaccinated)	Findings/Conclusion(s)
Blom et al., 2019 [32] Sweden	Zostavax vs. Unvaccinated	Retrospective population-based matched cohort study	99,189	≥50 years	Age	IR of HZ (per 100,000 person-years)	1.47	Overall VE was 34%; the greatest VE was in patients 61-75 and the only cohort to show a decrease in VZV reactivation compared to non-vaccinated individuals. VE generally decreased with age (except for the 61-75 age cohort).
Boutry et al., 2022 [33] International	Shingrix vs. Unvaccinated	LTFU study of clinical trials	7,277	67.2 years	Age	IR of HZ (per 1,000 person-years)	0.16	Overall, VE (84%) remained high for at least 7 years post-vaccination.
Curran et al., 2021 [34] Germany	Zostavax vs. Unvaccinated	LTFU study of clinical trials	~35,500,000	≥50 years	Age & years since vaccination	VE & approximate number of HZ cases prevented	NA	VE at time 0 was 98.9% and decreased by 1.5% each year for ages 50-69. VE was 95.4% and decreased by 2.3% each year for ages ≥70. The estimated number of HZ cases prevented was 884K, 603K, and 538K in three age cohorts: 50–59, 60–69, and ≥70, respectively.
Curran et al., 2021 [35] International	Shingrix vs. Unvaccinated	Observational retrospective study on placebo-controlled clinical trials	26,976	68.8 years	Assessment of frailty	VE	NA	VE was above 90% for all frailty subgroups (non-frail, pre-frail, and frail), greatly reducing the risk of VZV reactivation.
Dagnew et al., 2021 [36] International	Shingrix vs. Unvaccinated	Post hoc analysis of multinational clinical trial	29,305	68.8 years (Shingrix); 69.4 years (Placebo)	Age	VE and IR of HZ (per 1,000 person-years)	0.1	Shingrix was highly efficacious (90.5% overall) in those with immune-related comorbidities with no significant incidence of adverse effects compared to the placebo group.
Kim et al., 2021 [37] International (Asia only)	Shingrix vs. Unvaccinated	Post hoc analysis of two clinical trials (divided by age groups ≥50 years and ≥70 years)	2,723 in aged 50+ trial; 2,729 in aged 70+ trial	62.0 years (Both Shingrix and Placebo)	Age & years since vaccination	VE	NA	VE was 95.6% in the age 50+ trial and 94.7% in the age 70+ trial; VE % showed no consistent pattern of increase or decrease in yearly follow-ups.
Lu et al., 2021 [38] United States of America	Shingrix vs. Unvaccinated	Retrospective observational cohort study	4,842,579	≥50 years	Age, gender, race/ethnicity, and history of previous vaccination	IR of HZ (per 1,000 person-years)	0.33	Overall VE was 89.1% for HZ ophthalmicus, with VE highest in females, Hispanics, those with a previous history of HZ vaccination, and those aged 50-59; VE gradually decreased in each succeeding age cohort.
Matthews et al., 2018 [39] United Kingdom	Zostavax vs. Unvaccinated	Retrospective matched cohort study	295,135	70-79 years	None	IR (per 1,000 person-years) & VE	0.37	Overall, VE was 65.3%.
Sun et al., 2021 [40] United States of America	Shingrix vs. Unvaccinated	Retrospective cohort study	78,356	≥50 years	Age, gender, race/ethnicity	IR (per 100,000 person-years)	0.31	Overall VE was 83.5%, with the highest VE in Asians, Hispanics, males, those with a previous history of the Zostavax vaccine, and those aged 50-59.
Sun et al., 2021 [41] United States of America	Shingrix vs. Unvaccinated	Retrospective claims-based cohort study	4,769,819	≥50 years	Age, gender, race/ethnicity, insurance type, geographic region	IR (per 100,000 person-years) and VE	0.29	Overall, VE was 85.5%, with VE of 86.8% in ages 50-79 and 80.3% in ages 80 and older.
Walker et al., 2018 [4] United Kingdom	Zostavax vs. Unvaccinated	Population-based cohort study	487,901	≥50 years	Gender, vaccination cohort (routine vs. catch-up), history of zoster and time since vaccination	IR (per 1,000 person-years) and VE	0.35	Overall VE was 64%, which gradually waned each year after vaccination; lower VE (47%) in those with a history of HZ. Slightly lower VE in women (60%) than men (66%).
Willer et al., 2019 [42] International	Shingrix vs. Unvaccinated	Post hoc analysis of two international randomized clinical trials (divided by age groups 50+ and 70+)	14,753 in age ≥50 trial; 16,606 in age ≥70 trial	62.3 years (in age ≥50 years vaccinated), 62.2 years (in age ≥50 years placebo), 75.5 years (in age ≥70 years both vaccinated and placebo)	Age, gender, geographic region, ethnicity	VE	NA	Shingrix was highly efficacious, with >90% VE in men and women in both trials, highest in Europe in the ≥50 trial and in Asia/Australia in the ≥70 trial.
Oostvogels et al., 2019 [43] International	Shingrix vs. Unvaccinated	Pooled post hoc analysis of two parallel randomized, observer-blind, controlled trials	Only pooled total from both trials reported: 13,881 vaccinated cohort; 14,035 placebo cohort	Mean age pooled from both trials: 68.5 years in both vaccinated groups and placebo groups	Type of comorbidity	Prevalence (%), HZ IR (per 1,000 person-years), VE	Mean IR among all comorbidity categories (vaccinated to unvaccinated): 0.09	Shingrix had an overall high VE (>84% in all categories) for patients with many different types of comorbidities; VE was lowest in patients with respiratory disorders (84.5% (95% CI: 46.4–97.1)) and highest in patients with coronary heart disease (97.0% (95% CI: 82.3–99.9)). Renal disorders were the only medical conditions in which VE was not statistically significant (VE: 86.6% (95% CI: −4.5–99.7)).
Amirthalingam et al., 2018 [44] England	Zostavax vs. Unvaccinated	Population-based study	Mean number of participants (across several years): 310,001	60-89 years	Age, region, gender, time period, and vaccine eligibility; routine or catch-up vaccination; years since vaccination	IRR (projected to actual outcome), VE	NA	HZ incidence decreased by 35% (IRR 0.65 (95% 0·60–0·72)) in the first 3 years of vaccination for the three routine cohorts, and the equivalent decrease in the four catch-up cohorts was 33% for HZ (IRR 0.67 (0·61–0·74)). Overall, the VE against HZ was approximately 62%.
Baxter et al., 2018 [45] United States of America	Zostavax vs. Unvaccinated	Continuous prospective cohort study	~1,400,000	≥50 years	Age, years since vaccination, immune compromise status	VE, HR, IR (per 1,000 person-years)	0.65	VE against HZ was 49.1% (95% CI: 47.5, 50.6) overall across all follow-up participants. VE was 67.5% (95% CI: 65.4, 69.5) 1 year post-vaccination, which decreased to 47.2% (95% CI: 44.1, 50.1) in year 2 post-vaccination, which continued to decrease throughout the 8 years recorded in this study, with year 8 showing a VE of 31.8% (95% CI: 15.1, 45.2). VE in both participants ≥80 and younger participants was about the same; VE in immunocompromised participants was similar to VE in immunocompetent participants.

As expected from previous studies, Shingrix consistently had a higher VE than Zostavax. Most studies also agreed that VE waned with time, regardless of the type of vaccine administered. Many studies showed that younger age groups (i.e., individuals between 50 and 59 years of age) generally had higher VE than older age groups (individuals between 70 and 79 years of age and/or 80 years of age or older), with several studies showing a gradual decline within each increasing age group. Meanwhile, there was no consistent pattern of higher VE in any race/ethnicity or gender.

Table 3 shows the efficacy of zoster vaccines in preventing reactivation of VZV and long-term PHN. One study showed a PHN risk reduction from 15.4% to 9.2% in vaccinated individuals [7]. Another study conducted in Europe showed a 50% decrease in cases of PHN over the course of a three-year vaccination program [44]. Of the studies reported in Table 3, the majority found vaccination to decrease PHN rates, with one study even showing a VE against PHN of 100% in individuals 50 years of age or older [37]. Two studies also found vaccination to be slightly more effective for individuals under 70 years of age [7,37]. A decline in immune response, which has been shown to commonly occur with age, may be contributing to the slightly decreased VE among older adults. Although several studies compared VE between males and females, none of them showed a significant difference in VE based on sex [42].

**Table 3:** Studies analyzing VE and comparing the incidence of PHN in vaccinated versus non-vaccinated individuals aged 50+ aRR: adjusted risk ratio; HZ: herpes zoster; IMD: index of multiple deprivation; IR: incidence rate; IRR: incidence rate ratio; NA: not applicable; PHN: post-herpetic neuralgia; VE: vaccine efficacy

Source & Country	Study Group(s)	Study Design	Study Size Number (n)	Mean Age and/or Age Range of Participants	Stratification(s)	Type of Data Reported	The ratio of PHN IR (Vaccinated to Non-vaccinated)	Conclusion(s)/Findings
Klein et al., 2019 [46] United States of America	Zostavax vs. Unvaccinated	Open cohort study	1,454,841	≥50 years	Age, gender, race/ethnicity, outpatient visit frequency, influenza vaccination, comorbidities & immune status	VE & HZ IR (per 100,000 person-years)	0.64	VE was 64.8% overall, gradually decreasing each year after vaccination; VE showed no significant difference with age or immune status.
Willer et al., 2019 [42] International	Shingrix vs. Unvaccinated	Post hoc analysis of two international randomized clinical trials (VE against PHN was only calculated in the age ≥70 trial)	16,606 in age ≥70 years trial	75.5 years (among age 70+ years trials in both vaccinated and placebo)	Age, gender, geographic region, ethnicity	VE	NA	VE against PHN was 91.5% in women, 83.3% in men, and highest in those living in North America (100%) and identifying with "Other Ancestry" (100%) (Defined as American Indian or Alaskan Native, Native Hawaiian or Other Pacific Islander, or a person of mixed heritage).
Bruxvoort et al., 2019 [7] United States of America	Zostavax vs. Unvaccinated	Prospective cohort study	1,515	≥60 years	Age, gender, level of rated pain (out of 10, 10 being the worst)	Risk of PHN (%), aRR. 10-point pain score	NA	Risk of PHN was 9.2% in vaccinated patients and 15.4% in unvaccinated patients (aRR= 0.594); 2.0% of vaccinated patients and 4.8% of unvaccinated patients reported pain ≥7 PHN (aRR=0.332); risk was lower in adults aged <70 years than those ≥70 years regardless of gender or vaccination status.
Kim et al., 2021 [37] International (Asia only)	Shingrix vs. Unvaccinated	Post hoc analysis of two clinical trials (divided by age groups 50+ and 70+)	2,723 in aged ≥50 trial; 2,729 in aged ≥70 trial	62.0 years (both Shingrix and placebo)	Age & years since vaccination	VE	NA	VE against PHN was 100% in the age 50+ trial and 89.8% in the age 70+ trial.
Matthews et al., 2018 [39] United Kingdom	Zostavax vs. Unvaccinated	Retrospective matched cohort study	295,135	70-79 years	None	VE & HZ IR (per 1,000 person-years)	0.3	VE against PHN was 71.6% overall.
Walker et al., 2018 [4] United Kingdom	Zostavax vs. Unvaccinated	Population-based cohort study	380,147	68-79 years	Gender, ethnicity, IMD status, time since vaccination, cohort (routine vs. catch-up), influenza vaccination, history of zoster	VE & HZ IR (per 1,000 person-years)	0.21	VE against PHN was 81% overall, with no significant differences in VE noted with any stratified demographics such as gender, ethnicity, etc.
Amirthalingam et al., 2018 [44] England	Zostavax vs. Unvaccinated	Population-based study	Mean across several years: 310,001	60-89 years	Age, region, gender, time period, vaccine eligibility, routine or catch-up vaccination, years since vaccination	IRR (projected to actual outcome), VE	NA	PHN incidence decreased by 50% (0.50 (0.38–0.67)) in the first 3 years of vaccination for the three routine cohorts, and there was a 38% decrease in the four catch-up cohorts (0.62 (0.50–0.79)). VE was about 70-88% against PHN.

The three studies featured in Table 4 regarding PHN pathophysiology overall implied that PHN is likely not caused by further viral reactivation and is rather a result of neurological damage with possible genetic risk factors.

**Table 4:** Studies addressing/relating to the theory of PHN being the manifestation of additional VZV reactivation GWASs: genome-wide association studies; MRI: magnetic resonance imaging; NRS: numeric rating scale; OR: odds ratio; PHN: post-herpetic neuralgia; SNPs: single nucleotide polymorphisms; ZAP: zoster-associated pain

Source & Country	Study Groups	Study Design	Study Size Number (n)	Mean Age and/or Age Range of Participants	Stratification(s)	Type of Data Reported	Conclusion(s)/Findings
Li et al., 2020 [6] China	Risk of PHN in patients with deficits in pain modulation pathways vs. control group	Prospective cohort study	24 patients with PHN; 23 control group	63.78 years in the PHN cohort; 60.73 years in the control cohort	None	Psychophysical tests, pain intensity (short form of the McGill pain questionnaire), and structural MRI data	PHN patients had higher levels of anxiety and depression compared to the control group; structural MRI data showed that PHN patients had much smaller gray matter volumes of the thalamus and amygdala than the control group, as well as abnormal patterns of functional connectivity within ascending and descending pain pathways. Thalamus volume was also found to be negatively correlated with pain intensity in PHN patients.
Choi et al., 2020 [47] South Korea	Severity of PHN pain in ZAP patients with lower dexamethasone dose (Group A: one-time 5 mg) vs. higher dexamethasone dose (Group B: intermittent repeated total 15 mg)	Prospective, double-blind, randomized controlled trial	21 in Group A (one-time 5 mg) cohort; 21 in Group B (intermittent repeated total 15 mg) cohort	Group A: 64.67 years Group B: 67.43 years	None	Pain intensity, measured by NRS score, reduction in pain severity (%), OR	Intermittent repeated epidural dexamethasone bolus total 15 mg (Group B) resulted in more reduced pain scores and a higher likelihood of complete remission in ZAP patients without any adverse effects than Group A. Intermittent repeated epidural dexamethasone administration (Group B) is safe and is the more effective approach for PHN treatment past the acute phase compared to Group A.
Nishizawa et al., 2021 [48] Japan	Genotype of patients with chronic pain and/or PHN vs. healthy control group	GWASs using whole-genome genotyping arrays	89 PHN patients, 191 chronic pain participants, 282 control group	Age range: 22-89 years; mean: 65.18 years among all cohorts	None	SNPs, position on gene, related gene, chromosome, genotype	The *PRKCQ* gene within the *ABCC4* gene may cause individuals to be more susceptible to chronic pain conditions, and the rs4773840 SNP region (also in the *ABCC4* gene) may cause susceptibility to PHN.

A 2020 study compared the subjective pain relief according to the numeric rating scale experienced by zoster-associated pain (ZAP) patients who received a continuous epidural infusion of local anesthetics with a one-time 5 mg dose of dexamethasone compared with intermittent repeated doses (15 mg total) of dexamethasone. The results revealed a much more reduced pain rating in ZAP patients who received intermittent repeated doses than those who received one-time doses [47]. The authors proposed the anti-inflammatory properties of dexamethasone as an instrumental factor in pain relief and nerve repair by decreasing edematous and cytotoxic responses [47]. Given that dexamethasone is classified as an immunosuppressant glucocorticoid, it is unlikely that PHN is caused by further viral reactivation since dexamethasone was not observed to produce any adverse effects in the study participants [47].

Another study published in 2020 sought to determine if PHN was associated with subcortical/cortical alterations in the brain using magnetic resonance imaging (MRI) data. The study found that PHN patients had significantly less gray matter volume of the thalamus and amygdala compared to the healthy control group, and thalamus volume was negatively correlated with pain intensity (rated using the short-form McGill pain questionnaire) in PHN patients [6]. Subjective ratings of the Present Pain Index also negatively correlated with the strength of functional connectivity between the periaqueductal gray and primary somatosensory cortex in PHN patients [6]. These results were consistent with previous findings that suggested anatomical changes of the thalamus to be involved in neuropathic pain, and they indicate that PHN patients have deficits in the ascending and descending pain modulation pathways that may contribute to the development of chronic neuropathic pain [6].

A 2021 genome study aimed to identify genetic variants associated with chronic pain and PHN. The researchers identified the *PRKCQ* gene as having a strong association with chronic pain conditions. They also discovered a single nucleotide polymorphism (SNP) within the *ABCC4* gene region known as rs4773840 that had a significant association with PHN [48]. The *ABCC4* gene encodes the ABCC4 protein, which is involved in multi-drug resistance and acts as a regulator of intracellular cyclic nucleotide levels and mediator of cyclic adenosine monophosphate (cAMP)-dependent signal transduction. The *ABCC4* gene may also play a role in increasing susceptibility to chronic pain-related conditions such as PHN [48]. The authors of the study expressed a need for further research with larger sample sizes to support their findings.

This scoping review had its limitations as well as its strengths; the most notable limitations included the exclusive search of the PubMed database and the restriction to five years of data for article selection. Future scoping reviews or other study designs discussing this topic would likely benefit from searching various databases and unpublished literature as well as using different filters, keywords/phrasing, and publication year ranges. Another strategy that may aid in future studies would be to combine keyword searches using Boolean operators (e.g., "shingles" AND "Shingrix") as opposed to individual keyword searches.

This study's most significant strength was its inclusion of articles with diverse settings, experimental groups, and patients with a wide range of characteristics. This aids in providing the most holistic and accurate depiction of the many factors that may influence the onset of HZ and PHN.

## Conclusions

The findings of this scoping review suggest that physicians should be cognizant of their patients' comorbidities, immunization history, medication usage, psychological well-being, lifestyle, and family history, as well as how these factors may put them at a higher risk of developing HZ and PHN. This review highlights examples for physicians to consider when interviewing patients for their medical history. Physicians should also inform eligible patients (aged 50 and older and/or immunocompromised) of the benefits of receiving a shingles vaccination and encourage them to do so. With patient consent, the vaccine should be administered sooner (within close range of the age of eligibility) rather than later, as it is then more likely to provide protection against HZ and PHN and reduce its severity in the event of a breakthrough infection. As seen in the limited number of sources found relating to the cause/factors behind PHN, the pathophysiology of PHN should be further explored to conclusively determine whether the condition is the manifestation of further VZV reactivation. A better understanding of its cause(s) can lead to further research in developing additional forms of management/treatment for PHN, particularly in atypical cases where PHN lasts several years or is lifelong. Given the inconsistent research found regarding the association between VZV reactivation and body fat percentage, additional studies should also be done on this topic to determine whether there is a significant correlation between these variables.

## Appendices

Search strategy

The following keywords/phrases were each searched individually on PubMed to locate articles: chickenpox, postherpetic neuralgia, reactivation stimuli, Shingrix, shingles, varicella zoster, "varicella zoster viral reactivation", Zostavax.

To expedite the article selection process, the following filters were applied through the PubMed website for each keyword search to find articles that were fully available and more likely to fit the inclusion criteria: Free full text, Books and Documents, Classical Article, Clinical Study, Clinical Trial, Clinical Trial Protocol, Clinical Trial, Phase I, Clinical Trial, Phase II, Clinical Trial, Phase III, Clinical Trial, Phase IV, Comparative Study, Controlled Clinical Trial, Corrected and Republished Article, Dataset, Electronic Supplementary Materials, English Abstract, Evaluation Study, Government Publication, Guideline, Historical Article, Multicenter Study, Observational Study, Practice Guideline, Pragmatic Clinical Trial, Randomized Controlled Trial, Research Support, American Recovery and Reinvestment Act, Research Support, N.I.H., Extramural, Research Support, N.I.H., Intramural, Research Support, Non-U.S. Gov't, Research Support, U.S. Gov't, Non-P.H.S., Research Support, U.S. Gov't, P.H.S., Research Support, U.S. Gov't, Review, Scientific Integrity Review, Technical Report, Twin Study, Validation Study, Humans, English, Adult: 19+ years, from 2018/1/1 - 2023/4/1.

The keyword searches "Shingrix" and "Zostavax" had the following additional filters in lieu of "Adult: 19+ years": "Age: Middle Aged + Age 45+ years", "Middle Aged: 45-64 Years", "Aged: 65+ Years", "80 and over: 80+ years". Note that these filters are not infallible, as some articles fitting the exclusion criteria were still found and manually excluded during the article review process.
